# Novel oncogene COPS3 interacts with Beclin1 and Raf-1 to regulate metastasis of osteosarcoma through autophagy

**DOI:** 10.1186/s13046-018-0791-6

**Published:** 2018-07-03

**Authors:** Fan Zhang, Taiqiang Yan, Wei Guo, Kunkun Sun, Shidong Wang, Xing Bao, Kuisheng Liu, Bingxin Zheng, Hongliang Zhang, Tingting Ren

**Affiliations:** 1 Musculoskeletal Tumor Center, Peking University People’s Hospital, No. 11 Xizhimen South Street, Beijing, 100044 China; 2 Beijing Key Laboratory of Musculoskeletal Tumor, Beijing, 100044 China; 3 Department of Pathology, Peking University People’s Hospital, Beijing, 100044 China

**Keywords:** COPS3, Raf-1, Beclin1, Autophagy, Osteosarcoma metastasis

## Abstract

**Background:**

Expression of COP9 signalosome subunit 3 (COPS3), an oncogene overexpressed in osteosarcoma, has been demonstrated to be significantly correlated with tumor metastasis. However, the underlying mechanism by which COPS3 promotes metastasis of osteosarcoma and its role in autophagy remain unknown.

**Methods:**

The expression of COPS3 was detected in primary osteosarcoma tissues and matching lung metastasis tissues by immunohistochemistry (IHC). The effect of COPS3 on the metastasis of osteosarcoma cells was investigated by transwell, wound healing assays and animal studies. Indicated proteins was analyzed by western blotting when COPS3 was knockdown or overexpressed. The COPS3 Interacting protein was determined by immunoprecipitation assay. The relationship between COPS3 and autophagy was detected by western blotting and immunofluorescence.

**Results:**

We found that knockdown of COPS3 significantly reduced the lung metastasis of osteosarcoma cells in a mouse model, coinciding with downregulation of mitogen-activated protein kinase (MEK) and extracellular signal-regulated kinase (ERK) signaling. The silencing of COPS3 also inhibited the epithelial–mesenchymal transition (EMT) through the 90 kDa ribosomal S6 kinases (RSK), a family of signal transduction proteins downstream of MEK/ERK. Reciprocal immunoprecipitation assays revealed that COPS3 directly interacts with Raf-1, an upstream regulator of MEK/ERK. Surprisingly, Beclin1, an important autophagic protein, appeared in the COPS3-immunoprecipitates, along with the autophagic markers LC3-I and LC3-II. Loss of COPS3 completely inhibited H_2_O_2_-induced autophagic flux and reduced Beclin1 expression. Additionally, autophagy inhibitor or silencing of Beclin1 both decreased cell metastasis.

**Conclusions:**

Taken together, these data reveal a novel function of COPS3 in the regulation of autophagy and highlight the relationship between autophagy and metastasis in osteosarcoma cells.

**Electronic supplementary material:**

The online version of this article (10.1186/s13046-018-0791-6) contains supplementary material, which is available to authorized users.

## Background

As the most common primary malignancy of the bone, osteosarcoma can occur in 10- to 25-year-old adolescents and young adults and has a high malignancy and poor prognosis because of its metastatic tendency. Pulmonary metastasis is the major cause of death for osteosarcoma patients [1]. Although advances have been made in therapeutics for osteosarcoma, pulmonary metastasis remains the major obstacle to improve the prognosis for osteosarcoma patients [2]. Therefore, researchers have turned to the study of pathogenesis and transfer mechanisms, hoping to find specific genes that contribute to osteosarcoma metastasis [3]. While these genes could be used as early predictors of metastasis to guide clinical treatments and evaluate the prognosis, they could also be used as new therapeutic targets to improve the survival rate. Recently, a new oncogene, COPS3, was discovered and revealed to be involved in metastasis of osteosarcoma [4, 5].

COPS3 is located in chromosome region 17p11.2, which was demonstrated to be amplified in osteosarcoma by comparative genomic hybridization (CGH) [6]. The amplification of region 17p11.2 suggests that the oncogenes within it may play important roles in osteosarcoma [7]. COPS3 is one of the oncogenes found to be most consistently overexpressed after amplification [8]. COPS3 protein is a subunit of the COP9 signalosome (CSN), which exerts de-ubiquitination and protein kinase activities in a variety of processes [9]. Our previous study demonstrated that the frequency of COPS3 overexpression was significantly higher in osteosarcoma patients with metastasis than in patients without metastasis, and COPS3 silencing decreased the migration ability of osteosarcoma cells [4]. These findings indicate that overexpression of COPS3 might promote the metastasis of osteosarcoma. However, the function of COPS3 has not been established in vivo, and its specific molecular mechanism is still unclear.

Metastasis is a complicated and multi-step process in which tumor cells invade neighboring tissues, intravasate into the systemic circulation, extravasate to distant organs and proliferate there [10]. Through several studies, numerous factors and processes were found to be involved in the regulation of tumor metastasis, including the MAPK/ERK signaling pathway, EMT and matrix metalloproteinases (MMPs) [11]. MMPs are a group of proteolytic enzymes associated with extracellular matrix (ECM) protein degradation and have been implicated in tumor invasion [12]. EMT is a cellular program by which cells lose epithelial properties and gain mesenchymal properties, which contributes to enhanced cell migratory and invasive abilities [13]. The MAPK/ERK pathway also regulates the expression of proteases and genes involved in cellular attachment, formation of the cytoskeleton and cell survival, which are essential for metastasis [14].

Macroautophagy (hereafter referred to as autophagy) is a constitutive catabolic process that regulates degradation of unnecessary cytoplasmic constituents and damaged organelles [15]. Through this mechanism, cells eliminate dysfunctional, and potentially dangerous, molecules or organelles to maintain cellular homeostasis [16]. Additionally, autophagy is utilized as a cellular strategy to survive various environmental stresses, such as hypoxia, starvation, oxidative stress and drug stimulation [17–19]. A growing body of evidence indicates that autophagy also participates in the regulation of various aspects of metastasis, including cell motility and invasion, EMT, resistance to anoikis and immune evasion, as well as establishing the pre-metastatic niche [20]. Beclin1 is a critical regulator of autophagy and is involved in the formation of the autophagosome through the Beclin1-Vps34-Vps15 complex [21]. Although recent studies have shown that CSN is involved in the regulation of autophagy [22, 23], the regulatory role of COPS3 in autophagy remains unknown.

In the present study, we found that COPS3 interacts with Raf-1 to activate ERK/RSK signaling and subsequently induces EMT and expression of MMP9. Both in vitro and in vivo experiments demonstrated that COPS3 loss suppressed metastasis to the lung of osteosarcoma cells. Unexpectedly, we observed that COPS3 co-immunoprecipitated with both LC3 and Beclin1. Silencing of COPS3 completely inhibited H_2_O_2_-induced autophagic flux, decreased the expression of Beclin1 and inhibited cellular migration. Moreover, autophagy inhibitor or depletion of Beclin1 significantly inhibited the metastasis of osteosarcoma cells in transwell assays.

## Methods

### Chemicals and antibodies

U0126 (S1102) and BI-D1870 (S2843) were obtained from Selleck Chemicals (Houston, TX, USA). H_2_O_2_ (323381), chloroquine diphosphate salt (CQ; C6628) and antibody against LC3 (L7543) were purchased from Sigma-Aldrich (St. Louis, MO, USA). Antibodies against p-MEK1/2 (Ser217/221, 9154), MEK1/2 (9122), p-ERK1/2 (Thr202/Tyr204, 4370), ERK1/2 (4695), E-cadherin (3195), N-cadherin (13116), Vimentin (5741), MMP-9 (13667), Beclin1 (4122) were purchased from Cell Signaling Technology (CST, Beverly, MA, USA). Antibodies of Raf-1 (sc-2027), GAPDH (sc-25,778), IgG (sc-227) were acquired from Santa Cruz Biotechnology (Santa Cruz, CA, USA). COPS3 (ab79698) antibody was purchased from Abcam (Burlingame, CA, USA). Actin (TA-09) antibody was attained from ZhongShanJinQiao Biocompany (Beijing, China). Antibodies for p-RSK1/2/3/4 (S221/227/S218/232, RLP0436), RSK1/2/3/4 (RLT4183) were purchased from Ruiying Biological (Suzhou, China). Alexa Fluor® 488 Goat anti-Rabbit IgG (H + L) (A-11034) was acquired from Thermo Fisher Scientific (Waltham, MA, USA)**.**

### Tissue samples, cell culture and establishment of stable cell lines

Formalin-fixed and paraffin-embedded specimens of primary osteosarcomas and matched lung metastasis lesions were acquired from the Musculoskeletal Tumor Center, Peking University People’s Hospital, Beijing, China. And the usage of tissue samples was approved by the center’s Ethics Committee. The osteosarcoma cell lines 143B and SAOS-2 were cultured in DMEM medium supplemented with 10% fetal bovine serum (FBS) and 1% antibiotics at 37 °C with 5% CO_2_. HOS cells were cultured in RPMI-1640 with 10% FBS. The 143B, HOS and SAOS-2 cell lines were acquired from the American Type Culture Collection (ATCC, Rockville, MD, USA). 143B and HOS cells stably knockdown of COPS3 were carried out by shCOPS3 lentivirus (Hanbio, Shanghai, China) infection following with 2 mg/ml puromycin (Thermo Fisher Scientific, A1113803) selection. The knockdown efficiency was confirmed by real-time RT-PCR and Western blotting. The COPS3 shRNA sequences were as following: sense strand 5′-GCACATTCGATATGCAACA-3′ and antisense strand 5′-TGTTGCATATCGAATGTGC-3′.

### Transient transfection

The siRNA specific for human COPS3 (sc-60,457), Beclin1 (sc-29,797) and control siRNA (sc-37,007) were purchased from Santa Cruz Biotechnology. For interference, siRNA transfection was performed using Lipofectamine 3000 (Life Technologies-Invitrogen, Carlsbad, CA, USA, L3000015) according to the manufacturer’s protocol. COPS3 overexpression was accomplished by adenovirus carrying COPS3 with C Terminal Flag and His tag (Vigene Biosciences, Rockville, MD, USA, VH848797).

### RNA extraction and real-time RT-PCR

The total RNA of osteosarcoma cells or tissues was extracted using TRIzol reagent (Invitrogen, 15,596–018) according to the manufacturer’s Instructions. cDNA was synthesized with the extracted RNA using PrimeScript RT reagent Kit (TaKaRa, Dalian, Liaoning, China, RR037A). Real-time RT-PCR was performed using SYBR Premix Ex Taq II (TaKaRa, RR820A) with an Applied Biosystems Fast Real Time PCR System. Primer sequences used for amplification were as follows: COPS3 forward 5′-CGCTATTCTCACAGGTTCAG-3′ and COPS3 reverse 5′-GCATCATAGGCTCCATTCTC-3′; GAPDH forward 5′-GCACCGTCAAGGCTGAGAAC-3′ and GAPDH reverse 5′-ATGGTGGTGAAGACGCCAGT-3′. The reactions were performed at 95 °C for 30s followed by 40 cycles of 5 s at 95 °C and 34 s at 60 °C.

### Western blotting

Whole cell lysates were prepared using cell lysis buffer (CST, 9803) supplemented with protease inhibitors while the subcellular fractionation was performed using NE-PER™ Nuclear and Cytoplasmic Extraction Reagents (Thermo Scientific, 78,833) according to the instructions. Proteins were separated on 8–13% SDS-PAGE gel and transferred to PVDF membranes. Then the membranes were incubated with appropriate primary antibodies overnight at 4 °C and horseradish peroxidase-conjugated suitable secondary antibodies for 1 h at room temperature. The protein bands were detected with enhanced chemiluminescence (Merck Millipore, Billerica, MA, USA, WBKLS0500).

### Immunoprecipitation

A suitable amount of corresponding antibody was added into the cytoplasmic、nuclear lysate or whole cell lysis solution and incubated at 4 °C for 3 h. Then further incubated for 1 h with Protein A-Agarose (Vigorous Biotechnology, Beijing, China, P007). The immune precipitates were washed three times with lysis solution followed by eluted with SDS loading buffer. The eluent was subjected to immunoblotting.

### Immunofluorescence analysis

143B cells were cultured on coverslips. After different treatments, cells were fixed with 4% paraformaldehyde and permeabilized with PBS containing 0.1% Triton X-100 and 0.5%BSA. Then cells were incubated with primary antibody diluted in PBS containing 0.1% TritoX-100 and 0.5% BSA for 1 h at room temperature. After being washed in PBS for three times, the cells were exposed to suitable secondary antibody in the presence of 0.5% BSA. Images were observed with a fluorescence microscope.

### Immunohistochemistry

Paraffin sections were deparaffinized in dimethylbenzene and rehydrated through graded ethanol. Then 3% hydrogen peroxide was used to quench endogenous peroxidase activity and antigens were retrieved by sodium citrate buffer in a 95 °C water bath. After being blocked with 10% goat serum for 1 h, sections were exposed to COPS3 antibody (Bioworld, St. Louis Park, MN, USA, BS1192) overnight at 4 °C followed by incubation with the secondary antibody for 30 min at 37 °C. Chromogenic reaction was performed with diaminobenzidine followed by counterstained with hematoxylin. Immunostaining was assessed by two independent pathologists. The intensity was scored as follows: 0 (negative); 1 (weak); 2 (moderate); and 3 (strong). The percentage of positive cells was defined as follows: 0 (< 5%); 1 (5–25%); 2 (26–50%); 3 (51–75%); and 4 (> 75%). And the final score was seen as a multiplication.

### Wound healing assays

Cells were seeded in 6-well plates and cultured to 90% confluence. Then a scratch was made across the plates using a pipette tip and isolated cells were removed with PBS. Images of the wound were captured after incubation for 24 h. The area of the wound was measured using Image J.

### Transwell assays

24-well transwell chambers coated with or without matrigel (Corning, NY, USA, 354480, 3422) were used to analysis cell migration and invasion. Cells suspended with serum-free culture medium were planted into the upper chamber while the medium containing 10% FBS as an attractant was added to the bottom chamber. After incubation for 24 h, cells left in the upper chamber were wiped off with cotton swabs. Cells penetrating tanswell chambers were fixed with methanol and stained with crystal violet. Then cell numbers in five random field of vision (×100magnification) were counted under microscope.

### Animal studies

Four to six weeks old female BALB/c nude mice were purchased from Vital River (Beijing, China). Each mouse was subcutaneously injected with 3 × 10^6^ 143B cells stably transfected with shCtrl or shCOPS3 lentivirus respectively. Four weeks later, the mice were sacrificed. The xenografts were collected and weighed subsequently. And the tumor tissues were arranged for real-time RT-PCR and western blotting analysis.

For experimental metastasis analysis, 2 × 10^6^ cells (shNC-143B or shCOPS3-143B) suspended in 100 μl PBS were intravenously injected to mice through tail vein. After 4 weeks, all mice were sacrificed. The lungs of mice were collected and the pulmonary metastatic nodules were counted. Metastatic lungs were fixed in formalin followed with paraffin embedding. Serial sections of lungs were attained and stained with Hematoxylin and eosin (H&E) to confirmed the pulmonary metastatic lesions under microscope.

All animal experiments were approved by the Institutional Animal Care and Use Committee of Peking University People’s Hospital, Beijing, China.

### Statistical analysis

The normally distributed data were presented as mean ± standard deviation. The differences between two or more groups were analyzed by unpaired t test or one-way ANOVA. IHC scores of paired primary osteosarcoma and lung metastasis tissues were analyzed by Wilcoxon signed-rank test. A *P*-value< 0.05 was considered to have significant differences.

## Results

### Elevated expression of COPS3 in lung metastasis of osteosarcoma

**Fig. 1:**
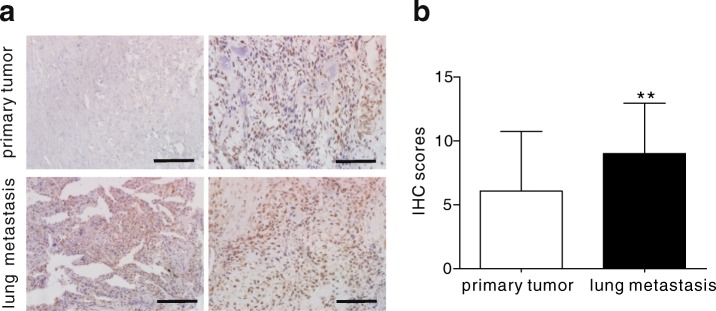
Elevated expression of COPS3 in lung metastasis of osteosarcoma. **a** COPS3 expression in 18 pairs of primary osteosarcoma tissues and matched lung metastasis tissues was evaluated by immunohistochemistry. **b** IHC scores of COPS3 staining in primary osteosarcoma and matched lung metastasis tissues were analyzed by Wilcoxon signed-rank test. **P < 0.01 vs. the primary tumor group. Scale bars: 100 μm

Our previous study indicated that osteosarcoma patients with overexpression of COPS3 had a higher risk of lung metastasis [4]. However, it remains unknown whether the expression of COPS3 was different between primary osteosarcomas and matched lung metastasis tissues. To better understand the relationship between the expression of COPS3 and osteosarcoma metastasis, 18 pairs of primary osteosarcoma tissues and matching lung metastasis tissues were collected for immunohistochemistry (IHC) analysis. The results demonstrated that lung metastasis tissues had significantly higher IHC scores than those of primary osteosarcomas (Fig. 1a and b), suggesting that osteosarcoma cells with high expression of COPS3 are likely to metastasize.

### COPS3 interacts with Raf-1 and activates MEK/ERK signaling

**Fig. 2:**
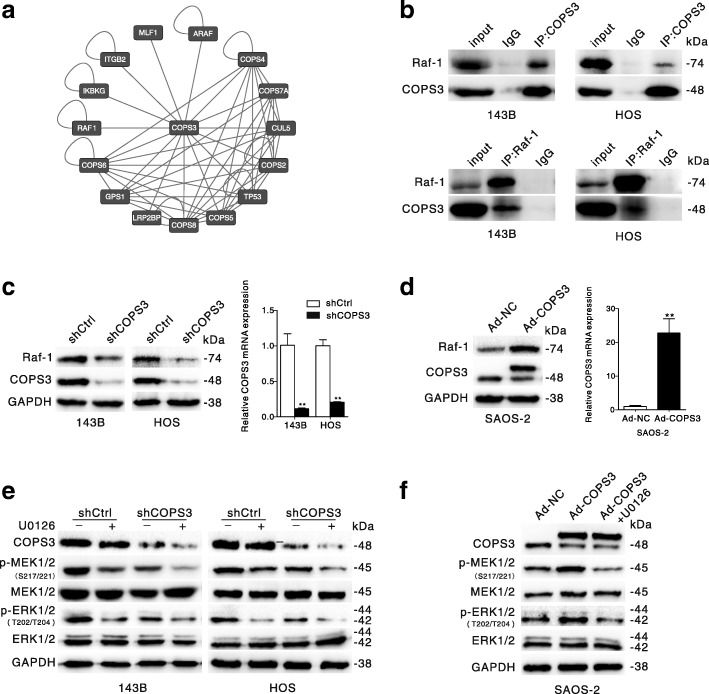
COPS3 interacts with Raf-1 and activates MEK/ERK signaling. **a** The COPS3 related protein–protein interaction network based on Human Protein Reference Database was constructed using Cytoscape. **b** 143B or HOS cells lysates were precipitated with COPS3 or Raf-1 antibody followed by immunoblotting probing with the indicated antibodies. **c** 143B and HOS cells were stably transfected with shCOPS3 or shCtrl lentivirus, COPS3 expression was analyzed by western blotting and Real-time RT-PCR, Raf-1 expression was determined by immunoblotting. **d** SAOS-2 cells were transfected with COPS3 adenovirus, the COPS3 level was analyzed by western blotting and Real-time RT-PCR, Raf-1 expression was determined by immunoblotting. **e** 143B and HOS cells stably transfected with shCOPS3 or shCtrl were treated with or without MAPK/ERK inhibitor U0126 (1 μM) for 24 h. Indicated proteins were detected by immunoblotting. **f** SAOS-2 cells transfected with Ad-COPS3 were exposed to MAPK/ERK inhibitor U0126 (1 μM) for 24 h. Western blot assay was performed with the indicated antibodies. The data are presented as mean ± S.D. from three independent experiments. **P < 0.01 vs. shCtrl group (**c**) or the Ad-NC group (**d**)

To explore the underlying mechanism of COPS3 in the regulation of osteosarcoma metastasis, we searched the Human Protein Reference Database (HPRD) to find proteins that may interact with COPS3 (Fig. 2a). We found that there was a high probability that COPS3 participates in the mitogen-activated protein kinases (MAPK) signaling pathway [4], and Raf-1 kinase was found to be a target protein likely to interact with COPS3. To test this prediction, we carried out co-immunoprecipitation studies. While Raf-1 appeared in COPS3 immunoprecipitates, the antibody against Raf-1 was also able to pull down COPS3 in both 143B and HOS osteosarcoma cell lines (Fig. 2b), confirming direct binding between COPS3 and Raf-1.

Since Raf-1 is an upstream regulator of MEK/ERK signaling, we next examined whether COPS3 plays a regulatory role in this signaling pathway. Stable knockdown of COPS3 in 143B and HOS cell lines was established via lentivirus-mediated silencing, while overexpression of COPS3 was accomplished by adenovirus-mediated infection in the SAOS-2 cell line, which has relatively low expression of COPS3 [4]. The efficiency of silencing and overexpression of COPS3 was confirmed by western blotting and real-time RT-PCR (Fig. 2c and d). While COPS3 depletion led to a decrease in the expression of Raf-1 in both 143B and HOS cells (Fig. 2c), its overexpression increased the level of Raf-1 in SAOS-2 cells (Fig. 2d). Moreover, COPS3 loss markedly inhibited the phosphorylation of MEK/ERK, which was further suppressed in the presence of U0126, a specific inhibitor of MEK (Fig. 2e). In contrast, overexpression of COPS3 increased the phosphorylation of MEK/ERK and U0126 inhibited this increase in MEK/ERK phosphorylation (Fig. 2f).

### COPS3 depletion reduces the migration and invasiveness of osteosarcoma cells

**Fig. 3:**
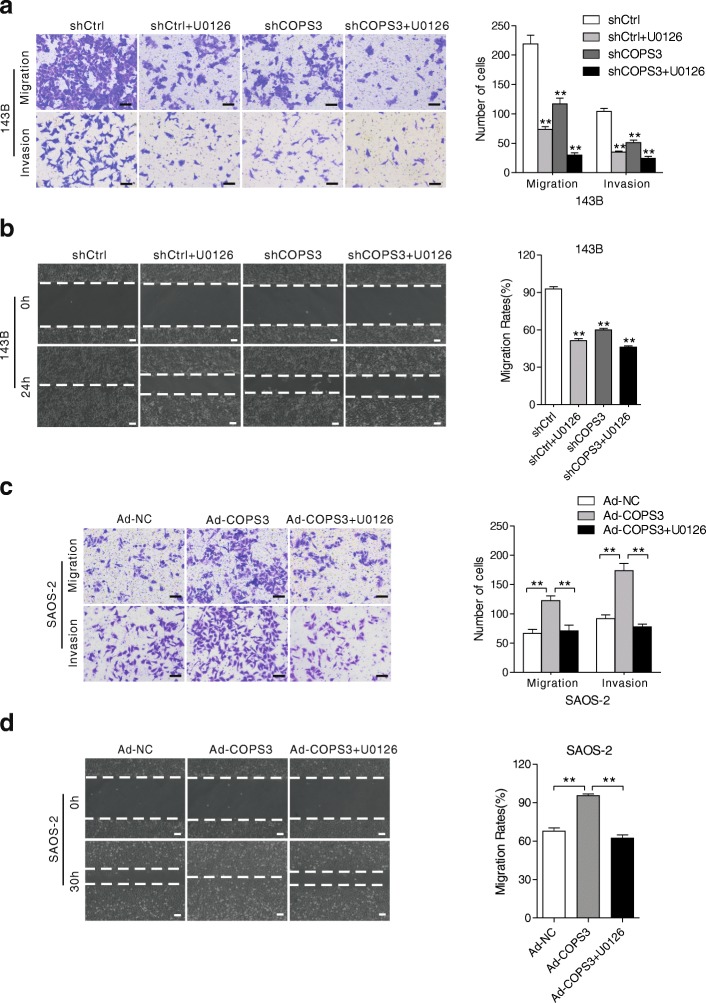
COPS3 enhances the migratory and invasive abilities of osteosarcoma cells. **a** and **b** 143B and HOS cells stably transfected with shCOPS3 or shCtrl were treated with or without MAPK/ERK inhibitor U0126 (1 μM) for 24 h, migratory and invasive abilities were evaluated by transwell assay (**a**). Wound-healing assay was also performed to analyze the migratory ability (**b**). **c** and **d** SAOS-2 cells transfected with Ad-COPS3 were exposed to MAPK/ERK inhibitor U0126 (1 μM) for 24 h, migration and invasion of the cells were analyzed by transwell (**c**) and Wound-healing assay (**d**). Scale bars: 100 μm. Each experiment was repeated three times, the data represented the mean ± S.D. **P < 0.01 vs. shCtrl group (**a** and **b**) or the Ad-NC group (**c** and **d**)

To determine whether COPS3 has a regulatory role in migration and invasion in cancer cells, transwell migration assays and wound-healing assays were performed. Compared with control shRNA (shCtrl), COPS3 depletion significantly inhibited cell migratory and invasive ability, which was further decreased in the presence of U0126 (Fig. 3a and b). Similar results were also obtained in HOS cells (Additional file 1: Figure S1 a and b). Overexpression of COPS3 increased the migration and invasion of SAOS-2 cells, whereas U0126 reversed COPS3-induced migration and invasion (Fig. 3c and d). These results suggest that COPS3 likely promotes the metastatic ability of osteosarcoma cells through the MEK/ERK/MAPK signaling pathway.

### COPS3 loss suppresses EMT through inhibition of RSK

**Fig. 4:**
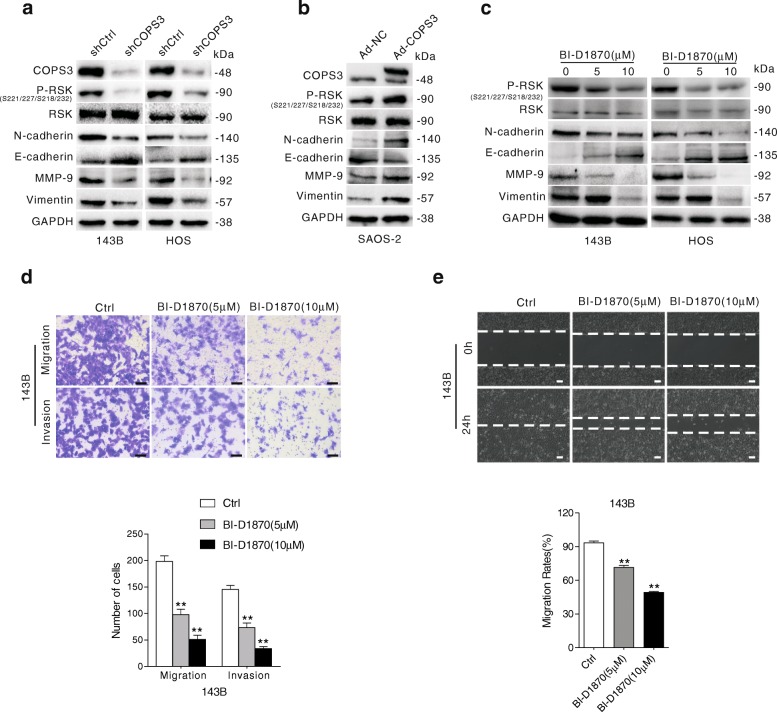
COPS3 loss suppresses EMT through inhibition of RSK (**a**) Western blotting analyzes the levels of RSK and its phosphorylated form, N-cadherin, E-cadherin, vimentin and MMP-9 in 143B and HOS cells stably transfected with shCOPS3 or shCtrl. **b** SAOS-2 cells were transfected with COPS3 adenovirus. The cell lysates were analyzed by immunoblotting with indicated antibodies. **c** Osteosarcoma cells were treated with 5 μM or 10 μM RSK inhibitor BI-D1870 for 24 h, cells lysates were subjected to immunoblotting using indicated antibodies. **d** and **e** 143B cells were exposed to 5 μM or 10 μM BI-D1870 for 24 h, migratory and invasive abilities were analyzed by transwell assay (**d**) and wound healing assay (**e**). Scale bars: 100 μm. Data representing mean ± S.D. were from three experiments with similar results, **P < 0.01 vs. control group

RSK is a member of the serine/threonine kinase family and can be activated by ERK/MAPK, and recent studies have revealed that RSK participates in the invasion and metastasis of cancer cell upon activation by RAS/ERK signaling [24, 25]. In 143B and HOS cells, we observed that knockdown of COPS3 suppressed the phosphorylation of RSK (Fig. 4a). In contrast, overexpression of COPS3 increased the phosphorylation of RSK (Fig. 4b), confirming our previous results that COPS3 plays a positive regulatory role in the Raf-1/MEK/ERK/RSK signaling pathway.

EMT is a cellular process that enables invasion to initiate tumor metastasis [13]. While knockdown of COPS3 increased the level of E-cadherin, an epithelial marker, its loss decreased the expression of vimentin and N-cadherin, both of which are indicative of mesenchymal characteristics (Fig. 4a). MMP-9, a member of the matrix metalloproteinase family and a critical player in tumor invasion, expression was also decreased in COPS3-depleted 143B and HOS cells (Fig. 4a). These results indicate that COPS3 loss inhibited EMT. Conversely, overexpression of COPS3 increased MMP-9 levels and induced EMT (Fig. 4b). To examine whether RSK is involved in EMT, BI-D1870, an inhibitor of RSK, was employed in both 143B and HOS cells. The inhibition of RSK inhibited EMT and decreased the expression of MMP-9 (Fig. 4c). Moreover, we observed that BI-D1870 decreased the number of migrating cells in the transwell assays and reduced the migration in wound-healing assays of the cells (Fig. 4d and e), indicating that RSK is involved in the process of both migration and invasion. Similar results were also obtained in HOS cells (Additional file 1: Figure S1 c and d). Given that COPS3 silencing inhibited EMT and ERK/RSK signaling, it is reasonable to believe that COPS3 mediates EMT via the ERK/RSK signaling pathway.

### COPS3 silencing suppresses the metastasis of osteosarcoma in vivo

**Fig. 5:**
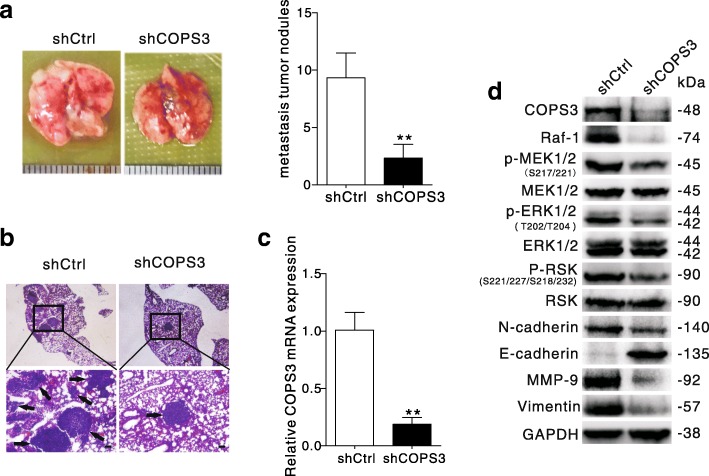
COPS3 silencing suppresses osteosarcoma metastasis in vivo. **a** and **b** Each group of mice were injected with 143B-shCtrl or 143B-shCOPS3 cells through tail vein (mean ± SD, *n* = 6). After four weeks, tumor nodules of lung metastasis were presented with representative images and H&E staining of lungs. **c** COPS3 expression in xenograft was detected by Real-time RT-PCR. **d** Variations of indicated proteins in vivo were analyzed by Western blotting. **P < 0.01 vs. shCtrl group. Scale bars: 100 μm

To validate the results obtained from in vitro experiments, we implemented a xenograft model of osteosarcoma in BALB/c nude mice. 143B cells stably infected with either shCtrl or shCOPS3 lentivirus were inoculated into mice. Although there was no significant difference in tumor size between the two groups after four weeks (Additional file 2: Figure S2 a and b), the shCOPS3 group exhibited significantly fewer metastatic tumor nodules compared with the shCtrl group (Fig. 5a and b). Knockdown with shRNA significantly decreased the expression of COPS3 at both the mRNA and protein level (Fig. 5c and d). Moreover, tissues from the shCOPS3 group expressed less MMP-9 and displayed inhibited Raf-1/MEK/ERK/RSK signaling. Similar to in vitro findings, COPS3 loss increased the level of E-cadherin and decreased both vimentin and N-cadherin levels, suggesting that it inhibited EMT (Fig. 5d).

### COPS3 is involved in the regulation of autophagy

**Fig. 6:**
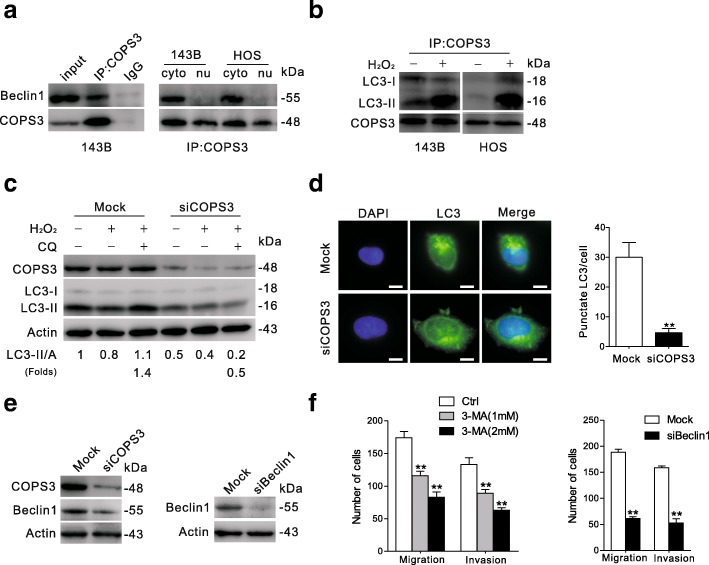
COPS3 inhibition diminishes the autophagic flux. **a** 143B cells lysates were co-immunoprecipitation with anti-COPS3 and probed with anti-Beclin1(left), cytoplasm extracts and nuclear extracts of 143B or HOS cells were separated and collected for co-immunoprecipitation (right). **b** 143B or HOS cells were exposed to 1 mM H_2_O_2_ for 4 h, the cell lysates were co-immunoprecipitation with anti-COPS3 and probed with anti-LC3-I/II. **c** 143B cells were transfected with siCOPS3 for 48 h, followed by treated with 1 mM H_2_O_2_ for 4 h in the presence or absence of CQ (8 μM). Indicated proteins were detected by immunoblotting and the relative levels of LC3-II were shown below. **d** Punctate LC3 of Mock and COPS3 silencing 143B cells were presented and quantified. Scale bars: 10 μm. **e** 143B cells were treated with COPS3 or Beclin1 siRNA. Indicated proteins expression were evaluated by immunoblotting. **f** 143B cells were exposed to 3-MA (1 and 2 mM) or Beclin1 siRNA. The migratory and invasive abilities were analyzed by transwell assays. Results above are expressed as the mean ± S.D. of three independent experiments. Scale bars: 100 μm **P < 0.01 vs. Mock or control group

Previous studies have demonstrated that both MEK/ERK and RSK play critical roles in the regulation of autophagy [26, 27], so we explored whether COPS3 participates in the regulation of autophagic process. Beclin1, a key player in autophagy [28], has been revealed to be a regulator of EMT [29]. Unexpectedly, we observed that COPS3 co-immunoprecipitated with cytoplasm-localized Beclin1 in 143B cells (Fig. 6a). Furthermore, we found that both LC3-I and LC3-II appeared in immunoprecipitates of COPS3 (Fig. 6b), and H_2_O_2_-induced autophagic stress enhanced the binding between LC3-II and COPS3 (Fig. 6b). COPS3 silencing completely inhibited H_2_O_2_-induced autophagic flux, as chloroquine (CQ) failed to cause LC3-II to accumulate upon H_2_O_2_ challenge (Fig. 6c) [30]. Immunofluorescence microscopy showed that punctate LC3 formation was significantly decreased in the COPS3-depleted cells (Fig. 6d), confirming that COPS3 silencing alone reduced the level of LC3-II (Fig. 6c). Moreover, we observed that expression of Beclin1 was decreased in COPS3-depleted cells (Fig. 6e). Treated with the autophagy inhibitor 3-Methyladenine (3-MA) or knockdown of Beclin1 significantly inhibited the migration and invasion abilities of cells in transwell assays (Fig. 6f), suggesting that autophagy participates in the metastasis of osteosarcoma cells.

## Discussion

COPS3 is a critical oncogene involved in the metastasis of osteosarcoma [4, 5, 8]. To further explore its specific molecular mechanism, in this study, we first demonstrate that COPS3 interacts directly with autophagic proteins and participates in the regulation of autophagy. Moreover, we found that COPS3 loss reduced the expression of Beclin1, through which COPS3 participates in regulating the migration of osteosarcoma cells.

Previous studies have revealed that COPS3 promotes osteosarcoma cell migration and invasion [31], and suppression of COPS3 could inhibit growth and induce apoptosis in lung cancer and hepatocellular carcinoma [32, 33]. However, our data clearly show that COPS3 silencing did not significantly inhibit osteosarcoma growth in vivo. Moreover, overexpression of COPS3 did not significantly enhance cell proliferation, although its overexpression markedly increased cell migration and invasion in osteosarcoma SAOS-2 cells [31]. Therefore, we reason that COPS3 might function in a cell type-dependent manner concerning its role in the growth of cancer cells, whereas it mainly acts to promote metastasis in osteosarcoma cells.

RSK plays a critical role in regulating motility and invasive ability of carcinoma cells [25]. Recently, a genome-wide RNAi screen identified several migration-regulating genes having RSK as a common and critical downstream effector [34]. RSK was found to regulate proliferation and promote metastasis in glioblastoma [35, 36]. In human breast cancer cells and tissues, the human endogenous retrovirus type K (HERV-K) Env protein mediated tumorigenesis and metastasis via the Ras/ERK/RSK pathway [37]. Consistent with these studies, we found that COPS3 promoted metastasis of osteosarcoma mainly through RSK signaling.

As a downstream of effector of Raf-1/MEK/ERK, RSK inhibition induced autophagy in oral cancer cells [26], whereas the ERK inhibitor PD098059 suppressed the autophagic process [38]. In colon cancer cells, Raf-1/ERK signaling could activate autophagy through stimulating the phosphorylation of G-alpha-interacting protein [39, 40]. Furthermore, the activation of Raf-1/MEK/ERK was revealed to enhance the Beclin1-dependent autophagy [41, 42]. Therefore, COPS3 is likely to induce autophagy through MEK/ERK signaling. Additionally, COPS3 was found to regulate both the phosphorylation of MEK/ERK and the expression of Beclin1 in the present study. Moreover, LC3-I, LC3-II and Beclin1 appeared in COPS3 immunoprecipitates, suggesting an intimate relationship between autophagy and COPS3.

For a long time, autophagy and EMT have been considered to be distinct, unrelated processes. However, recent studies indicate that these two important processes in cancer cells are closely linked and intricately related. Several studies reported a direct impact of autophagy on EMT regulation in carcinoma cells [43]. Much like its dual role in cancer, the effect of autophagy on EMT is controversial and likely depends on the cell type and/or stage of tumor progression [44, 45]. While the EMT process requires autophagy to support the viability of potentially metastatic carcinoma cells, a growing body of additional evidence indicates that autophagy prevents EMT and may induce reversion of the EMT phenotype in cancer cells [45–48]. It has been reported that autophagy is required for pulmonary metastasis of hepatocellular carcinoma (HCC) cells. Either Beclin1 or ATG5 silencing in HCC cells impaired the incidence of pulmonary metastases in a mouse model [44]. In contrast, autophagic induction leads to decreased migration and invasion in glioblastoma cells, whereas autophagy deficiency increased cell motility and invasiveness [45]. In A549 human lung adenocarcinoma cells, Beclin1 promotes apoptosis and reduces invasion [49], whereas knockdown of Beclin1 decreased invasion and metastasis in osteosarcoma cells [50]. Our data clearly show that Beclin1 silencing led to reduced migration of osteosarcoma cells, and thus, it is likely that the effect of Beclin1 on cancer cell metastasis is cell type dependent. Given that COPS3 silencing inhibited H_2_O_2_-induced autophagy, reduced Beclin1 expression and suppressed MEK/ERK phosphorylation, autophagy is likely to play a role in COPS3-mediated metastasis.

## Conclusions

**Fig. 7:**
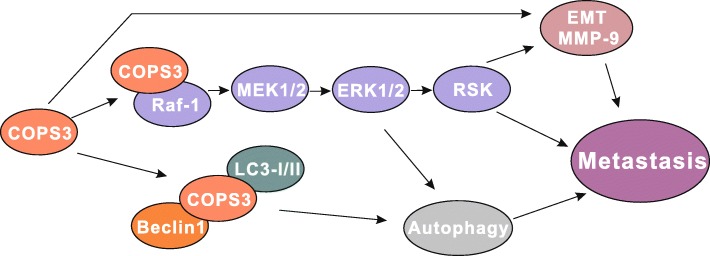
A schematic drawing of COPS3 regulating mechanism in osteosarcoma metastasis

In summary, our study revealed that COPS3 plays an important role in linking Raf-1/MEK/ERK signaling pathway and autophagic regulation (Fig. 7). It broadens our understanding of the regulatory role of autophagy in tumor cellular metastasis and identifies COSP3 as a potential therapeutic target for osteosarcoma treatment.

## Additional files

Additional file 1: **Figure S1.** Knockdown of COPS3 reduces the migratory and invasive abilities of osteosarcoma cells through ERK/RSK. (a and b) HOS cells stably transfected with shCOPS3 or shCtrl were treated with or without MAPK/ERK inhibitor U0126 (1 μM) for 24 h. Migratory and invasive abilities were evaluated by transwell assay and Wound-healing assay. (c and d) HOS cells were exposed to 5 μM or 10 μM RSK inhibitor BI-D1870 for 24 h. Transwell assay and wound-healing assay were performed to assess migratory and invasive abilities. The data are presented as mean ± S.D. from three independent experiments. Scale bars: 100 μm. **P < 0.01 vs. shCtrl group (a and b) or the control group (c and d). (PDF 1338 kb)

Additional file 2: **Figure S2.** COPS3 downregulation did not significantly affect tumorigenesis of osteosarcoma cells. (a) 143B-shCtrl and 143B-shCOPS3 cells were subcutaneously injected to BALB/c nude mice. The xenografts were collected four weeks later. (b) the tumor weights were compared between 143B-shCtrl and 143B-shCOPS3 group. (mean ± SD, *n* = 4). The ns was short for no significant. (PDF 172 kb)

## Abbreviations

3-MA: 3-Methyladenine
COPS3: COP9 signalosome subunit 3
CQ: Chloroquine.
EMT: Epithelial–mesenchymal transition
ERK: Extracellular signal-regulated kinase
IHC: Immunohistochemistry
MAPK: Mitogen-activated protein kinases
MEK: Mitogen-activated protein kinase
MMPs: Matrix metalloproteinases
RSK: 90 kDa ribosomal S6 kinases
