# Supplementary figures and images for: Novel oncogene COPS3 interacts with Beclin1 and Raf-1 to regulate metastasis of osteosarcoma through autophagy
Source: J Exp Clin Cancer Res. 2018 Jul 3;37:135. doi: 10.1186/s13046-018-0791-6 (PMC6029018; doi:10.1186/s13046-018-0791-6)

a

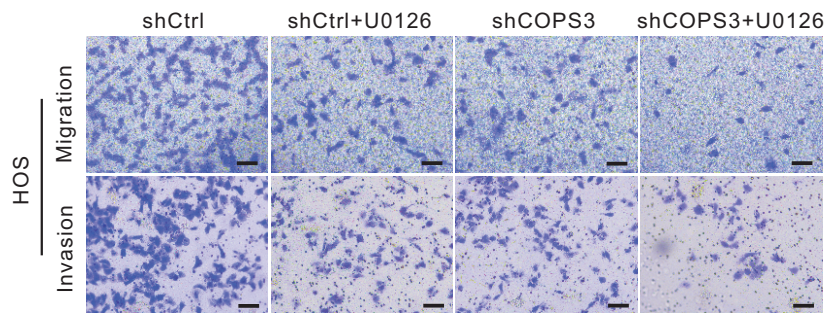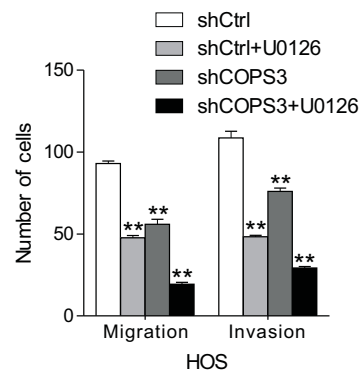

b

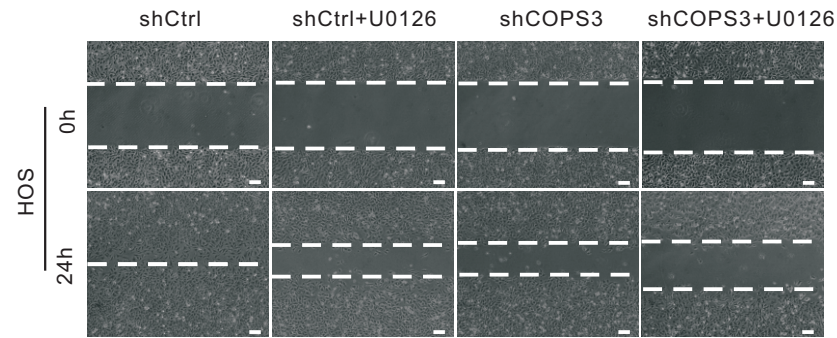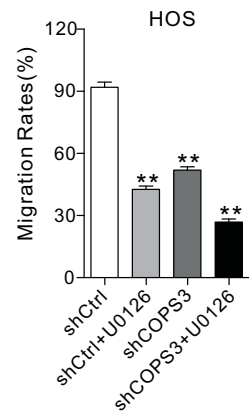

c

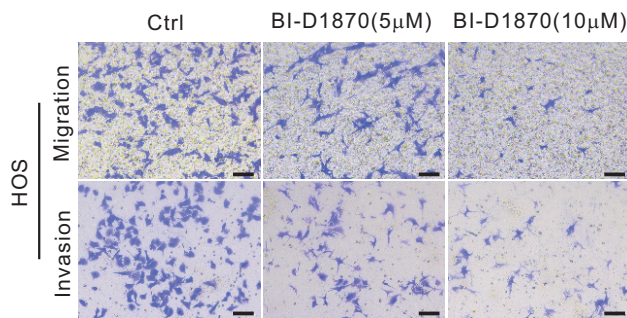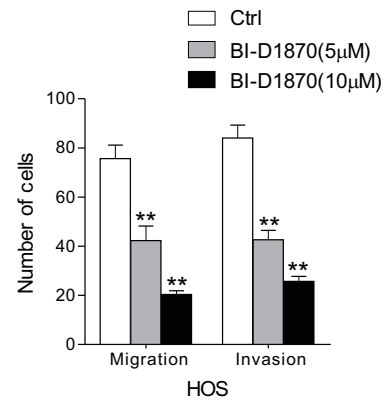

d

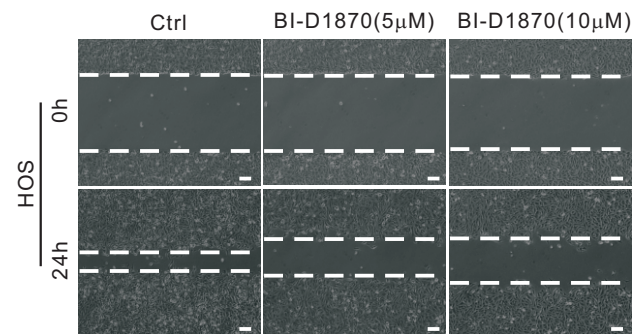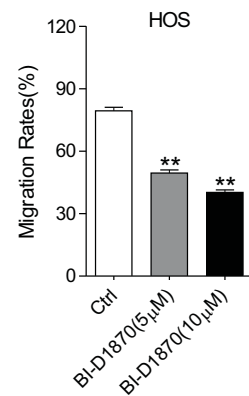

Supplement: Supplementary file 1 — Figure S1. Knockdown of COPS3 reduces the migratory and invasive abilities of osteosarcoma cells through ERK/RSK. (a and b) HOS cells stably transfected with shCOPS3 or shCtrl were treated with or without MAPK/ERK inhibitor U0126 (1 μM) for 24 h. Migratory and invasive abilities were evaluated by transwell assay and Wound-healing assay. (c and d) HOS cells were exposed to 5 μM or 10 μM RSK inhibitor BI-D1870 for 24 h. Transwell assay and wound-healing assay were performed to assess migratory and invasive abilities. The data are presented as mean ± S.D. from three independent experiments. Scale bars: 100 μm. **P < 0.01 vs. shCtrl group (a and b) or the control group (c and d). (PDF 1338 kb) [file 13046_2018_791_MOESM1_ESM.pdf]

**a**

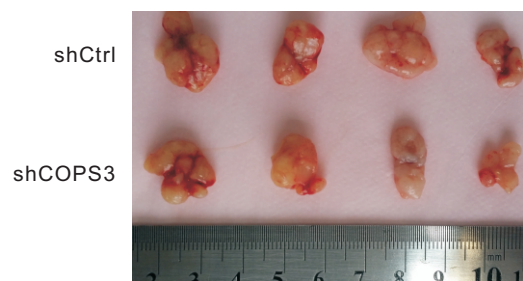

**b**

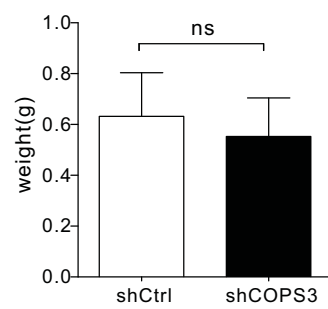

Supplement: Supplementary file 2 — Figure S2. COPS3 downregulation did not significantly affect tumorigenesis of osteosarcoma cells. (a) 143B-shCtrl and 143B-shCOPS3 cells were subcutaneously injected to BALB/c nude mice. The xenografts were collected four weeks later. (b) the tumor weights were compared between 143B-shCtrl and 143B-shCOPS3 group. (mean ± SD, n = 4). The ns was short for no significant. (PDF 172 kb) [file 13046_2018_791_MOESM2_ESM.pdf]
